# Schizophyllum commune

**DOI:** 10.3201/eid2803.211051

**Published:** 2022-03

**Authors:** Monika Mahajan

**Affiliations:** Postgraduate Institute of Medical Education and Research, Chandigarh, India

**Keywords:** Schizophyllum commune, fungi, clamp connection, Basidiomycetes, basidiocarps, basidiospores, split-gill mushroom, wood-rotting fungus, Elias Magnus Fries, Hans Kniep

## Schizophyllum commune [skiz-of′-ǐ-ləm kom′-yoon]

**Figure 1:**
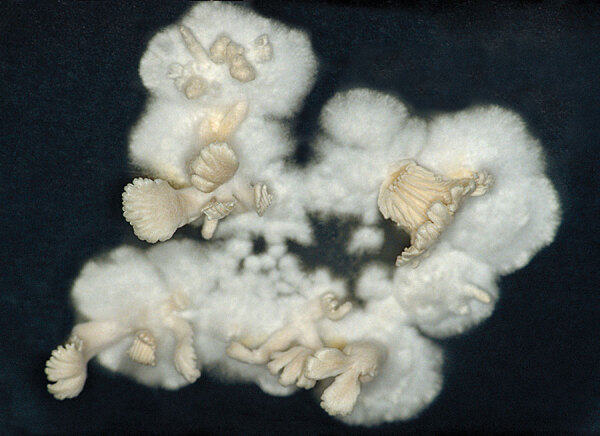
Colony of *Schizophyllum commune* on a culture plate. Numerous sexual reproductive structures, or fruiting bodies, called basidiocarps can be seen. Note the split gills. Source: https://phil.cdc.gov/Details.aspx?pid=307

**Figure 2:**
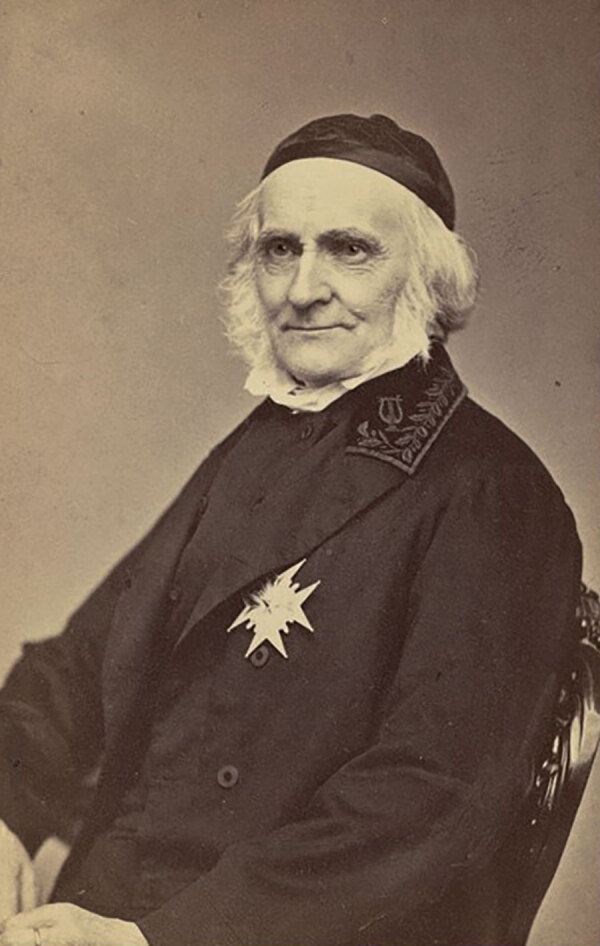
Swedish mycologist Elias Magnus Fries (1794–1878), who assigned the scientific name to *Schizophyllum commune.* Photograph by Emma Schenson, 1865. Source: Kungliga Biblioteket, Stockholm LIBRIS, Elias Fries, https://www.kb.se

*Schizophyllum commune* or split-gill mushroom, is an environmental, wood-rotting basidiomycetous fungus (Figure 1). *Schizophyllum* is derived from “*Schíza*” meaning split because of the appearance of radial, centrally split, gill like folds; “*commune*” means common or shared ownership or ubiquitous. Swedish mycologist, Elias Magnus Fries (1794–1878), the Linnaeus of Mycology, assigned the scientific name in 1815 (Figure 2). German mycologist Hans Kniep in 1930 discovered its sexual reproduction by consorting and recombining genomes with any one of numerous compatible mates (currently >2,800).

Isolation by Kligman in 1950 of fleshy fungus that had fan-shaped sporophores from a case of onychomycosis was regarded as interesting. However, it was dismissed as improbable because mushrooms were not known to invade animal tissue. This emerging fungal pathogen is characterized by the presence of clamp connections, hyphal spicules, and formation of basidiocarps with basidiospores.
